# Psychotherapists' Perspectives and Support Needs in Treating Patients With Disabilities: Results From an Online Survey

**DOI:** 10.1002/cpp.70159

**Published:** 2025-09-30

**Authors:** Bastian Hardt, Merit Donauer, Kristina Dürr, Johannes Michalak

**Affiliations:** ^1^ Department of Psychology and Psychotherapy Witten/Herdecke University Witten Germany

**Keywords:** accessibility, mental health services, outpatient psychotherapy, persons with disabilities

## Abstract

**Objectives:**

Persons with disabilities are at elevated risk for mental disorders, yet little is known about the experiences, attitudes and needs of psychotherapists regarding this population. This study is among the first to examine outpatient psychotherapists' experiences in treating patients with disabilities and to explore their perspectives on accessibility and therapists' support needs.

**Design:**

An exploratory cross‐sectional online survey was conducted in collaboration with a regional statutory health association.

**Methods:**

A total of *N* = 371 licensed outpatient psychotherapists in Germany participated in the study. All were qualified to treat adult patients under statutory health insurance. The survey included self‐constructed items assessing treatment experiences, perceived accessibility and support needs, as well as a questionnaire for measuring explicit attitudes towards disability.

**Results:**

Most participants (*n* = 340; 91.6%) reported prior experience treating patients with disabilities. Attitudes were generally positive and significantly associated with the quality—but not the quantity—of treatment experience. Psychotherapists perceived greater efforts in treating patients with intellectual and hearing impairments, and 357 (96.2%) identified barriers in their own outpatient settings. Only 93 (25.1%) considered themselves experts in disability‐related topics. A strong demand for further training was expressed, particularly for working with specific types of impairments, with a preference for professional development programmes offered both online and in person.

**Conclusions:**

The findings highlight the need for enhanced training, improved structural accessibility and informed policy development. Despite positive attitudes, notable gaps remain in preparedness and environmental access, emphasizing the importance of integrating disability‐related content into psychotherapist training and professional development.

## Summary

- Most psychotherapists in outpatient care already have experience treating patients with disabilities, but the reported patient numbers indicate that this group remains underserved.
- Psychotherapists generally hold positive attitudes towards patients with disabilities, which are linked more to positive treatment experiences than to the sheer amount of prior contact.
- Accessibility in outpatient psychotherapy settings is still perceived as limited, with major barriers including communication difficulties, a lack of accessible materials and inadequate practice infrastructure.
- Targeted professional training is strongly requested, especially for working with patients with hearing and intellectual impairments.
- Improving disability‐related content in training and continuing education is crucial to ensure inclusive psychotherapy.

## 1 Introduction

According to the United Nations Convention on the Rights of Persons with Disabilities (CRPD) (UN General Assembly 2007), ‘persons with disabilities include those who have long‐term physical, mental, intellectual, or sensory impairments which, in interaction with various barriers, may hinder their full and effective participation in society on an equal basis with others’. The CRPD (UN General Assembly 2007) highlights existing disparities in healthcare access for persons with disabilities and emphasizes the need for barrier‐free services and ongoing training in healthcare systems. Ensuring both structural and content‐related accessibility in psychotherapy and psychiatry is therefore crucial to improving equitable care. As reported by the World Health Organization (WHO, n.d.), approximately 1.3 billion people, or 16% of the global population, live with significant disabilities. This number is expected to rise significantly by 2050. Within the European Union, 27% of the population over the age of 16, equating to approximately 101 million people, reported having some form of disability in 2023 (Council of the European Union 2023). Concurrently, persons with disabilities are considered vulnerable populations, studies showing an increased risk of developing mental health issues or mental disorders compared with persons without disabilities (Brunes et al. 2019; Buckles et al. 2013; Jiang et al. 2020; Mazza et al. 2020; Shoham et al. 2019; Zheng et al. 2017).

High prevalence rates of disabilities and the limited availability of specialized psychotherapists mean that psychotherapists in general practice also come into contact with patients with various types of impairments. However, the experiences and specific needs of non‐specialized psychotherapists in this context have received little academic attention to date. Such insights are essential for a comprehensive understanding of how to design future barrier‐free structures and inclusive services in clinical psychology.

Reliable cross‐national comparisons of psychotherapist density remain difficult because of variations in data collection methods, professional classifications and health system structures. However, Germany, where this study was conducted, reports one of the highest densities in Europe, with approximately 48 psychotherapists per 100,000 inhabitants in 2022 (Organization for Economic Co‐operation and Development, OECD 2022). This disparity reflects significant regional differences in access to mental health services across Europe. For further context, in Germany, there were 39,627 licensed psychotherapists in outpatient care for adults in 2023 (Kassenärztliche Bundesvereinigung [National Association of Statutory Health Insurance Physicians] 2024). In comparison, publicly accessible directories from self‐help associations list only 49 specialized therapists or counsellors for patients with hearing impairments and 34 for those with vision impairments (Deutscher Blinden‐ und Sehbehindertenverband, n.d.; Deutscher Gehörlosen‐Bund, n.d.).

Beyond existing gaps in service provision, little is known about how psychotherapists perceive their work with persons with disabilities—both in general and with regard to specific types of impairments. Only a small number of studies have addressed this topic, highlighting challenges such as therapists' feelings of unpreparedness and difficulties in providing accessible care. Studies exploring therapists' personal reflections, such as Dovidio et al. (2011), revealed that unconscious biases may subtly impact therapeutic outcomes when working with patients with disabilities. Chacón et al. (2013) explored therapists' experiences working with persons with intellectual disabilities, finding that many therapists felt both professionally and emotionally challenged by this work. Besika et al. (2018) investigated therapists' attitudes towards persons with learning disabilities. The findings indicate that therapists who lack specific training in working with this client group may make errors in the therapeutic process. On the other hand, education, professional experience and direct personal contact with persons with learning disabilities contribute to more positive attitudes. For instance, Olkin et al. (1996) reviewed psychologists' experiences and found that many professionals reported a lack of training and preparedness to address the specific needs of patients with disabilities. Similarly, Critchfield (2002) emphasized the necessity of specialized training to effectively communicate and work with patients who are deaf or deafblind, pointing out that many psychotherapists lack direct experience with such populations. Steinlechner et al. (2017) investigated the accessibility of psychotherapeutic practices in Germany and identified significant gaps in accommodating individuals with physical and sensory disabilities, noting that therapists often desired additional support and training. Research on psychotherapists indicates that (1) professional contact and experience and (2) education and training are two key factors shaping attitudes, self‐efficacy expectations and perceived competence in treating patients with disabilities. Additionally, research from other social and medical professional fields highlights that contact with persons with disabilities is a significant predictor of positive attitudes towards this population (Alnahdi et al. 2020; Page and Islam 2015; Shields and Taylor 2014).

International and European disability advocacy organizations have consistently highlighted the need for accessible and inclusive psychotherapy. They emphasize addressing systemic barriers, including structural, environmental and attitudinal challenges, while offering practical recommendations for improving accessibility. The European Disability Forum (EDF) underscores that inaccessible health services, both physical and digital, remain a significant hurdle for persons with disabilities. In their *position paper*, EDF (2022) calls for comprehensive accessibility audits in mental health facilities and increased investment in assistive technologies to reduce disparities in care. Similarly, Inclusion Europe focuses on the needs of persons with intellectual disabilities, highlighting the lack of adapted environments in mental health services. Their *2021 report* advocates for the adoption of universal design principles, such as simplified navigation systems, pictogram‐based signage and accessible communication materials (Inclusion Europe 2021). Mental Health Europe (MHE) has taken a proactive approach in addressing these issues directly with mental health professionals. Their *2020 guide for professionals* offers concrete strategies to foster inclusion, including staff training on disability awareness, adapting therapeutic methods for diverse needs and ensuring the accessibility of therapeutic settings (MHE 2020).

The current literature underscores the urgent need for expanded mental health services tailored to persons with disabilities. These findings highlight the necessity for further empirical research to develop barrier‐free and inclusive psychotherapy. Despite these calls, little is known about the experiences, attitudes and needs of psychotherapists in general practice who are not specialized in treating patients with disabilities and who work in outpatient settings (Hardt, Heidenreich, et al. 2025a). In light of these gaps, this study is, to our knowledge, the first to examine the explicit attitudes and support needs of non‐specialist psychotherapists working in outpatient settings towards patients with disabilities, using a targeted recruitment strategy aimed at ensuring representativeness. We explored how many psychotherapists in outpatient settings have treated this population and how they evaluate their experiences—both with patients with disabilities in general and with regard to specific types of impairments (i.e., physical, intellectual, hearing and vision impairments). Based on previous research (e.g., Besika et al. 2018; Chacón et al. 2013), we hypothesized a positive association between professional contact with persons with disabilities and psychotherapists' attitudes towards them. To identify potential areas for improvement, our online survey also assessed the current state of accessibility and perceived support needs (e.g., disability‐related training, information and resources) among outpatient psychotherapists, with a primary focus on the German and Central European mental health systems. In accordance with existing policy (e.g., the CRPD, UN General Assembly 2007) and research demands (e.g., Hardt, Heidenreich, et al. 2025a; Hardt, Graser, et al. 2025b) for inclusive psychotherapy, we further expected high reported levels of treatment‐related barriers and treatment‐related effort, as well as limited self‐assessed knowledge (i.e., academic and psychotherapy training) and substantial informational needs regarding disability‐related topics (i.e., forms of professional development). Although there is evidence for the effectiveness of psychotherapy for persons with disabilities, particularly intellectual disabilities (Graser et al. 2022), substantial challenges in dissemination and implementation remain.

## 2 Method

The current study was approved by the Ethics Committee of [University Name Removed for Review] (Ethics Application No. S‐231/2021) and was conducted in accordance with national law and the tenets of the Declaration of Helsinki of 1975 (as revised). Informed consent was obtained from all participants.

### 2.1 Participants

This study was conducted in Germany, where adult psychotherapy is practiced by trained psychologists or physicians. The profession is regulated by the Psychotherapist Act (Psychotherapeutengesetz, PsychThG) (Deutscher Bundestag 2019). Licensed psychotherapists can provide treatment under public health insurance if trained in approaches such as psychoanalysis, psychodynamic therapy, cognitive behavioural therapy or systemic psychotherapy.

For a targeted recruitment strategy, we collaborated with the Kassenärztliche Vereinigung Nordrhein (KVNO), the statutory association responsible for licensed psychotherapists and physicians practicing in outpatient settings in parts of North Rhine‐Westphalia, a federal state of Germany. This approach ensured that only licensed psychotherapists actively practicing in outpatient settings were contacted, providing access to a well‐defined and representative sampling frame for this professional group. Upon request, the KVNO reported a total of 2569 full‐time listed psychotherapists, with a mean age of 52.73 years and 78.2% being female, who are eligible to treat adults in outpatient care (Kassenärztlichevereinigung Nordrhein, KVNO [Association of Statutory Health Insurance Physicians and Psychotherapists of Northrhein] n.d.). This number represents roughly 6.5% of all licensed psychotherapists in Germany. All 2569 psychotherapists registered with the KVNO were invited to participate in the online survey via an email sent by the KVNO, including a cover letter from the research team. Two reminder emails were distributed at 4‐week intervals. As no personal data were collected, reminders were sent to all psychotherapists regardless of prior participation.

The study included a total number of *N* = 371 participants with a mean age of 52.78 (SD = 10.64; 77.1% female). By a total number of 2569 psychotherapists, this corresponds to a response rate of 14.4%. Additional sample characteristics including information on demographics are presented in Table S1 (Supporting Information S1).

### 2.2 Measures

The online survey consisted of two questionnaires. Because of the absence of established instruments for measuring experiences and ratings related to psychotherapeutic work with persons with disabilities, a self‐developed questionnaire was created (Tables S1 and S2 in Supporting Information S2). The self‐developed questionnaire comprised 24 items across the following areas: (1) demographic information (e.g., age, gender and psychotherapeutic approach), (2) professional experiences with persons with disabilities (e.g., treating or not treating patients with disabilities and details on the type of impairment), (3) perceived accessibility in psychotherapy (e.g., experienced and anticipated barriers and treatment‐related effort) and (4) support needs (e.g., disability‐related knowledge and needs for further training and formats). At the beginning, the questions addressed persons with disabilities in general and subsequently specified the following types of impairment: (1) physical, (2) intellectual, (3) hearing and (4) vision impairments. The response format was adapted to the content of each question. For example, the construct of professional contact with persons with disabilities was assessed with the question: ‘Have you had professional experience as a psychotherapist with patients who have physical, intellectual, or sensory impairments?’ and a dichotomous response option (Yes/No) for the categories: persons with disabilities in general and for the four specific types of impairments. Quality of the treatment experience was assessed using the question: ‘How would you describe your general experiences with persons with disabilities in the context of psychotherapy?’ The response options for these items were presented on a 5‐point Likert scale (1–5): ‘*very negative*’, ‘*rather negative*’, ‘*mixed*’, ‘*rather positive*’ and ‘*very positive*’. The first author and two graduate students (B. Sc. degree in psychology) pretested the survey items. They assessed the comprehensibility and face validity of the items, and modifications were made where necessary.

For the second questionnaire, the current study used the Fragebogen zur Messung der expliziten Einstellungen zu Behinderung (*Questionnaire for Measuring Explicit Attitudes toward Disability*, EXPE‐B, Schröter et al. 2019) to assess psychotherapists' attitudes towards persons with disabilities (Tables S3 and S4 in Supporting Information S2). The instrument can be used across different professional groups and aims to capture the mechanism of Othering, which refers to the perception of persons with disabilities as ‘the others’. The EXPE‐B consists of 16 items rated on a 6‐point Likert scale (0–5), anchored by ‘*strongly disagree*’ and ‘*strongly agree*’. It includes two subscales: (1) Structural Discrimination (10 items; maximum score: 50; e.g., ‘The issue of prejudice against people with disabilities is often exaggerated.’) and (2) Personal Contact (6 items; maximum score: 30; e.g., ‘I would be happy if my child had children with disabilities as close friends.’). A total attitude score is calculated by summing both subscales, with a maximum possible score of 80. The validation of the questionnaire was conducted with a sample of prospective teachers. The participants' mean total score was 67.52 (SD = 8.37). The questionnaire demonstrates good reliability (Cronbach's *α* = 0.78, test–retest reliability of *r* = 0.81). In the present study, the total score was used, where a higher score indicates a more positive attitude towards persons with disabilities.

### 2.3 Procedure

Data collection was conducted online using LimeSurvey. Accessing the online survey, we used an open format without personalized URLs, allowing participation only from psychotherapists contacted by the KVNO. At the beginning of the survey, participants were provided with information about the study's procedures, voluntariness and data protection measures.

### 2.4 Data Analysis

Data analysis was conducted using IBM SPSS Statistics software (Version 28). Only fully completed survey responses were considered. We used descriptive statistics to characterize the sample. Independent‐sample *t*‐tests were conducted to compare the group means of psychotherapists with and without professional contact to patients with disabilities. Pearson correlations were calculated to examine associations between the EXPE‐B attitude score and ratings for treatment experience, accessibility and effort in treatment. For all analyses, the significance level was set at *p* < 0.05.

Some psychotherapists reported extremely high numbers of patients treated per year and across their entire career (e.g., 4000 per year), which are unlikely to be plausible in outpatient psychotherapy settings. Since no empirically validated or universally accepted cutoff exists for realistic caseloads in this field, we applied a data‐driven approach to reduce the influence of extreme values. Descriptive statistics are therefore reported both for the full sample and for a reduced sample including only values below the 95th percentile (cf. Wilcox 2017). Additionally, medians are provided for all relevant variables, as they offer robust estimates of central tendency in skewed distributions and are less affected by outliers (Erceg‐Hurn and Mirosevich 2008).

## 3 Results

The following section presents the results, beginning with the attitude towards persons with disabilities for the sample of 371 psychotherapists.

### 3.1 Attitude Toward Persons With Disabilities

In the current sample of psychotherapists, the total EXPE‐B attitude score towards persons with disabilities was *M* = 69.84 (SD = 7.44; *N* = 371). This was significantly higher than the mean score reported in the validation study by Schröter et al. (2019), which was based on a sample of prospective teachers (*M* = 67.52, SD = 8.37, *N* = 308). An independent‐sample *t*‐test confirmed this difference, *t*(677) = 3.82, *p* < 0.001, with a small effect size (*d* = 0.29).

### 3.2 Professional Contact to Patients With Disabilities

Of the 371 psychotherapists, 340 (91.6%) reported prior professional contact in treating patients with disabilities, whereas 31 (8.4%) had no such experience. In contrast to our expectations, attitudes of psychotherapists with prior experience (*M* = 69.84, SD = 7.40) did not significantly differ from those without such experience (*M* = 69.94, SD = 8.07), *t*(369) = 0.07, *p* = 0.943. The effect size of this difference was negligible (*d* = 0.01). Among the 340 psychotherapists who reported prior professional contact with patients with disabilities, the most frequently treated types of impairments were physical impairments (*n* = 307; 90.3%), followed by intellectual impairments (*n* = 237; 69.7%), hearing impairments (*n* = 202; 59.4%) and vision impairments (*n* = 162; 47.6%).

### 3.3 Quality of Treatment Experience

Based on prior professional contact, the 340 psychotherapists evaluated the quality of treatment experiences with patients with disabilities in general and for the four specific types of impairments (Table 1). The most positive experience ratings were reported for treating patients with physical or vision impairments, whereas ratings were less positive for intellectual and hearing impairments. Moreover, the reported quality of treatment experiences was positively and significantly correlated with the EXPE‐B attitude score, except in the group of patients with intellectual impairments. More positively rated treatment experiences were associated with more positive attitudes towards persons with disabilities.

**TABLE 1:** Ratings for the quality of treatment experiences and Pearson correlations with the EXPE‐B attitude score.

	Therapists with experience *n*	Quality of treatment experience *M* (SD)	Correlation with the EXPE‐B score *r*
Patients with disabilities in general	340	3.89 (0.72)	0.23**
Physical impairment	314	4.06 (0.67)	0.24**
Intellectual impairment	255	3.40 (1.00)	0.10
Hearing impairment	213	3.58 (0.87)	0.23**
Vision impairment	182	4.09 (0.69)	0.30**

* *p* ≤ 0.05.

** *p* ≤ 0.01.

### 3.4 Number of Patients With Disabilities

Not all 340 psychotherapists with prior professional contact with patients with disabilities provided complete data on the number of patients with and without disabilities. All available responses were analysed, and separately a trimmed sample including only values within the 95th percentile to account for extreme outliers.

In the full dataset, the number of patients with disabilities treated per year ranged from 0 to 300 (*M* = 10.90, SD = 30.16, *Mdn* = 3; *n* = 339), and the total number across the psychotherapists' careers ranged from 1 to 5000 (*M* = 82.77, SD = 337.48, *Mdn* = 12; *n* = 338). In the trimmed sample, the annual number ranged from 0 to 50 (*M* = 6.32, SD = 9.38, *Mdn* = 2; *n* = 322), and the career total ranged from 1 to 400 (*M* = 31.29, SD = 55.00, *Mdn* = 10; *n* = 322). The percentage of patients with disabilities among all patients treated per year was on average *M* = 7.92% (SD = 12.09%), with a median of *Mdn* = 3.85% in the full dataset. Within the trimmed sample, the mean was *M* = 5.70% (SD = 5.80%), and the median was *Mdn* = 3.73%. Further details on the treatment frequency are presented in Table S2 (Supporting Information S1).

### 3.5 Accessibility of Outpatient Psychotherapy

One or more barriers in outpatient treatment for patients with disabilities were reported by 357 (96.2%) of the 371 psychotherapists, whereas only 14 (3.8%) saw no barriers in their professional practice. Across the full sample, the most frequently reported barriers were communication difficulties (e.g., verbal and non‐verbal communication; *n* = 297; 80.1%), building‐related limitations (e.g., stairs, narrow hallways or doorways, restroom access and acoustics; *n* = 233; 62.8%) and challenges related to therapeutic content and procedures (e.g., conducting diagnostics, using therapy materials and implementing tasks or exercises; *n* = 191; 51.5%). Additional barriers included difficulties in initially contacting a therapist (e.g., inaccessible via phone, messenger or email; *n* = 113; 30.5%) and issues related to the route to therapy (e.g., lack of access to public transport and limited or inaccessible parking; *n* = 95; 25.6%). A small number of participants reported other barriers in open‐text responses (e.g., time constraints and difficulty finding an available therapist; *n* = 13; 3.5%).

All 371 psychotherapists evaluated (a) the expected treatment‐related effort for working with patients with disabilities compared with those without disabilities, (b) the accessibility of their personal work setting and (c) the expected accessibility of general outpatient psychotherapy services for patients with disabilities in Germany (Table 2). The highest ratings for treatment‐related effort were reported for patients with intellectual and hearing impairments. Accessibility in the participants' personal work settings was rated more positively than the expected accessibility of the general outpatient care system. Notably, ratings of treatment‐related effort and accessibility of general outpatient psychotherapy were significantly and negatively correlated with the EXPE‐B attitude score.

**TABLE 2:** Treatment‐related effort, accessibility of the personal work setting and general outpatient psychotherapy, including Pearson correlations with the EXPE‐B attitude score (*N* = 371).

	Rating *M* (SD)	Correlation with the EXPE‐B score *r*
Evaluation of the treatment‐related effort for patients with disabilities in general	3.73 (0.92)	−0.20**
Physical impairment	2.92 (1.18)	−0.16**
Intellectual impairment	3.89 (1.10)	−0.13**
Hearing impairment	3.90 (0.95)	−0.14**
Vision impairment	3.33 (1.06)	−0.11*
Evaluation of accessibility for the personal treatment setting in general	3.07 (1.05)	0.01
Physical impairment	3.16 (1.35)	0.01
Intellectual impairment	3.62 (1.10)	0.01
Hearing impairment	3.51 (1.14)	−0.03
Vision impairment	3.41 (1.01)	0.06
Evaluation of accessibility in general outpatient psychotherapy	2.30 (0.68)	−0.13*

* *p* ≤ 0.05.

** *p* ≤ 0.01.

### 3.6 Disability‐Related Knowledge and Support Needs

Participants' self‐assessed knowledge about persons with disabilities varied: 110 (29.6%) reported having basic knowledge (‘I have dealt with the topic little or not at all and do not recall learning about it during my training, studies, or school years. My understanding is therefore largely based on general knowledge.’), 168 (45.3%) indicated having professional knowledge (‘My knowledge is based solely on what I learned during my training, university studies, school education, and general knowledge.’) and 93 (25.1%) considered themselves experts in this field (‘As a psychotherapist, I have engaged professionally with various types of impairments and have gained insight into the living conditions of affected persons through direct contact.’).

Disability‐related topics were covered extensively in respondents' academic degree programs by 32 (8.6%), briefly by 106 (28.6%), not addressed in the curriculum by 190 (51.2%) and not remembered by 43 (11.6%). In psychotherapist training, disability‐related topics were covered extensively by 19 (5.1%), briefly by 109 (29.4%), not addressed in the curriculum by 210 (56.6%) and not remembered by 33 (8.9%).

A considerable number of psychotherapists reported a need for further training tailored to specific types of impairments: 120 (32.3%) for physical impairments, 221 (59.6%) for intellectual impairments, 212 (57.1%) for hearing impairments and 156 (42.0%) for vision impairments. In contrast, 75 participants (20.2%) indicated no information needs involving further training (Table S3 in Supporting Information S1). Regarding preferred types of support for treating patients with disabilities, 168 psychotherapists (45.3%) selected online professional development programmes, followed by 127 (34.2%) who indicated a need for more information about disability subcultures, 122 (32.9%) who favoured in‐person professional development opportunities and 109 (29.4%) who selected professional literature. Additionally, 83 respondents (22.4%) expressed interest in sign language courses, whereas 78 (21.0%) reported no need for further support (Figure S1 in Supporting Information S1).

## 4 Discussion

This study successfully reached a large number of psychotherapists and collected data on their experiences, assessments, attitudes and support needs regarding the treatment of persons with disabilities. These findings offer valuable insights for this emerging research field and bear practical implications for outpatient mental healthcare.

The results reveal that nine out of 10 psychotherapists already have experience in treating persons with disabilities. The proportion of patients with disabilities treated per year ranged from approximately 6% to 8%. Given the presence of statistical outliers, the median of slightly under 4% might represent a more accurate estimate. These figures suggest that the outpatient treatment of patients with various types of impairments is already occurring, as predicted by international and European developments (Council of the European Union 2023; WHO, n.d.), and that this treatment is being provided by therapists who are not specialized in treating patients with disabilities or working in specialized institutions. However, when compared with the proportion of persons with disabilities (27% in Europe, Council of the European Union 2023, and 9.3% in Germany, (Statistisches Bundesamt [Federal Statistical Office of Germany] 2024), these numbers clearly indicate an ongoing and significant undersupply of persons with disabilities in outpatient psychotherapy.

When comparing the distribution of psychotherapists' experience with different types of impairments to prevalence patterns in the general population, some notable discrepancies appear (Statistisches Bundesamt [Federal Statistical Office of Germany] 2024): As expected, physical impairments are highly represented, aligning with their high prevalence in official disability statistics (58%). Interestingly, intellectual or psychological impairments might be somewhat more frequently represented in therapeutic experience than their population‐based prevalence (15%) would suggest. In contrast, hearing (4%) and vision impairments (4%)—although comparably prevalent—might be underrepresented in terms of reported psychotherapeutic experience.

Compared with preservice teachers (Schröter et al. 2019), psychotherapists demonstrated significantly more positive attitudes towards persons with disabilities. Although this effect should be considered small, both samples overall reported very positive attitude scores. The contact hypothesis—which posits that increased contact leads to more positive attitudes (Alnahdi et al. 2020; Besika et al. 2018; Page and Islam 2015)—should be considered from two perspectives in the context of psychotherapy: Although therapists with prior experience treating patients with disabilities did not differ significantly in their overall attitudes compared with those without such experience, it is noteworthy that a more positive evaluation of these treatment experiences was associated with a significantly more positive attitude in general. This association was found across the evaluation of experiences for patients with disabilities in general and for all types of impairments, except for intellectual impairments. Thus, the results appear to support the notion that the quality of experiences, rather than the mere quantity of experience, may play a crucial role, as suggested by Dovidio et al. (2011) and Chacón et al. (2013).

The vast majority of therapists reported various barriers within their own work environment, highlighting, as also emphasized by Steinlechner et al. (2017) and the European Disability Forum (2022), the persistent shortcomings in outpatient mental healthcare for persons with disabilities. The evaluation of treatment‐related effort was significantly and negatively associated with therapists' attitudes towards persons with disabilities across all impairment types, suggesting that more positive attitudes may be linked to lower perceived barriers. At the same time, patients with intellectual and hearing impairments were rated as requiring the highest treatment‐related efforts. Possible explanations for this finding may include the need to adapt communication forms and therapeutic methods within therapy (Critchfield and Ehlers 2018; Hatton et al. 2021). Furthermore, therapists rated the accessibility of their personal treatment settings higher than that of general outpatient care.

Regarding disability‐related knowledge, only one in four psychotherapists described themselves as experts in this field, and only four out of 10 reported that disability‐related topics were addressed during their graduate courses or postgraduate psychotherapist training. These findings emphasize the continuing need, as advocated by various stakeholders (e.g., the CRPD, UN General Assembly 2007; researchers such as Critchfield 2002 and Olkin et al. 1996; and alliances like the European Society for Mental Health and Deafness [ESMHD, n.d.]), to strengthen the integration of disability‐related content in the education and training of mental health professionals. The need for further information and support was also clearly reflected in our results: A large number of therapists expressed interest in receiving further training on different types of impairments, particularly intellectual and hearing impairments. This might once more indicate the specific challenges involved in therapeutic adaptations when working with these two patient groups (Chacón et al. 2013; Critchfield 2002). It should also be noted that the reported need for information was lower regarding physical and vision impairments. Physical impairments encompass a broad range of highly diverse conditions affecting different parts of the body (WHO 2001), which may make it more difficult to establish a clear and consistent definition of this patient group compared with others (e.g., hearing or vision loss). The findings related to vision impairments are also noteworthy, as the full range of structural and content‐related barriers experienced by this group might still not be fully recognized within the field of clinical psychology (Hardt, Heidenreich, et al. 2025a; Hardt, Graser, et al. 2025b). The willingness to engage in various forms of support underscores the demand for professional development programmes, both online and in‐person, as well as for learning more about disability subcultures and alternative forms of communication (e.g., sign language). Only a small proportion of therapists reported no specific interest for additional training or support opportunities—a group that also showed significantly fewer positive attitudes towards persons with disabilities compared with those seeking further information. These findings highlight the importance of openness and knowledge‐seeking in shaping inclusive professional attitudes in the group of psychotherapists (Besika et al. 2018; Olkin et al. 1996).

In summary, on one side, our findings reveal prior therapeutic experience, generally positive attitudes towards persons with disabilities and a link to positive evaluations of treatment quality by psychotherapists. On the other side, the results show that relatively few patients with disabilities are currently being treated, various barriers exist in outpatient settings and critical perspectives on the current state of accessibility in service provision were expressed. Still, there is a strong openness towards working with this client group, and a clear and urgent need for training and support was articulated within this field.

### 4.1 Limitations

The present study provides valuable new insights into the outpatient psychotherapy of persons with disabilities. However, its methods and findings also have some limitations that should be mentioned when interpreting the results and guiding future research. Our study relied exclusively on self‐reported data from psychotherapists regarding a socially sensitive and highly relevant topic. This reliance raises the possibility of a social desirability bias, in which participants may have responded in a manner perceived as more socially acceptable, especially given that recruitment was conducted through a professional organization. However, no specific measurements of social desirability were included in the survey, which should be addressed in future studies. The sample itself might have been biased by the topics emphasized in the recruitment cover letter. A relatively high proportion of respondents reported experience in treating persons with disabilities, which could reflect a self‐selection effect based on prior interest or positive attitudes towards the subject matter.

In addition, the response rate among participants was only 14.4%, which may impact the generalizability of the findings and introduce potential bias. To address concerns about representativeness, we requested official KVNO statistics on all licensed outpatient psychotherapists, including key demographics such as age and gender. This enabled a direct comparison between our sample and the overall population. It is probable that psychotherapists who participated were more likely to have prior experience with patients with disabilities and to hold more open attitudes towards disability‐related topics. Nevertheless, as Taherdoost and Madanchian (2024) discuss, even surveys with lower response rates can yield valid and valuable insights, provided that the sample characteristics are adequately considered. Overall, our sample closely reflected the KVNO population on key demographics such as age and gender, which partially mitigates concerns about selection bias. Still, we cannot exclude the possibility that factors such as professional experience may have influenced participation, which should be considered when interpreting the findings.

Furthermore, the findings are based on experiences within the German healthcare system. Although it is likely that similar trends can be observed in other healthcare systems, differences in service provision, training standards and disability policies between countries suggest the need for caution when generalizing the results internationally. Future research should explicitly examine cross‐national similarities and differences in psychotherapy for patients with disabilities.

Finally, we focused primarily on general treatment experiences and attitudes towards persons with disabilities in general. Specific types of impairments, such as dual sensory impairments (e.g., deafblindness) or multiple impairments, were not separately assessed. Future studies may benefit from tailoring similar research designs to address particular types of impairments in more detail.

### 4.2 Implications

The findings of this study point to several implications for improving outpatient psychotherapy for patients with disabilities. A substantial number of psychotherapists expressed a clear desire for accessible sources of information, continuing education and opportunities for professional support. Although some foundational literature is already available to support self‐directed learning (e.g., Bron 2015; Glickman 2008; Olkin 2017), evidence‐based and scientifically evaluated programmes remain scarce.

Our findings underscore an urgent need for targeted professional development programmes—both online and in‐person—to build psychotherapeutic competencies in working with patients with disabilities. One example of an initiative addressing the intersection of disability and mental health is the *Training in Intellectual Disability and Mental Health for Frontline Staff* project, coordinated in Ireland (Salvador‐Carulla et al. 2013). Aimed at professionals working with adults with intellectual impairments and co‐occurring mental health conditions, the project developed modular training materials focusing on communication, assessment, treatment planning and service coordination. The conceptual foundations of such programmes should be further pursued and expanded to develop structured training formats tailored to various types of impairments, as reflected in the diverse information needs identified in our study. Such programmes should follow scientific quality standards, be supported by health policy and adopt participatory approaches involving both experienced clinicians and persons with lived disability experience. Yet a review of available training opportunities reveals a striking gap: Most existing programmes focus exclusively on intellectual impairments, whereas programmes for working with persons with physical, hearing or vision impairments are virtually non‐existent. This absence points to a severe structural neglect in current continuing education systems and underscores the urgent need to develop and systematically implement impairment‐specific training programmes in this field.

Our findings reinforce previous calls to move beyond individual expertise or personal interest and towards evaluated, research‐informed continuing education. Curricula at universities and psychotherapist training institutions should integrate disability‐related content more consistently and at an earlier stage. For example, the reform of the German Psychotherapist Act (PsychThG) (Deutscher Bundestag 2019) established a legal basis for this by requiring the inclusion of topics such as accessibility and disability competence in academic and clinical training. Yet, systematic implementation remains inconsistent. Clearly defined modules, practical experiences and qualified instructors are essential to ensure meaningful integration, as emphasized by advocacy groups and recent international guidelines (Mental Health Europe 2020; ESMHD, n.d.).

Given that the vast majority of participants in our study had prior experience treating patients with disabilities and simultaneously expressed a strong need for further information and training, there is a pressing need to establish structured support systems for psychotherapists in practice. In addition to formal continuing education, low‐threshold networks and centralized information platforms could facilitate peer exchange, provide curated access to evidence‐based resources and guide clinicians towards suitable training opportunities. International organizations such as the European Society for Mental Health and Deafness (ESMHD, n.d.) or the International Association for the Scientific Study of Intellectual and Developmental Disabilities (IASSIDD n.d.) have initiated interdisciplinary exchange formats such as workshops, conferences and continuing education opportunities in these domains. In contrast, a comparable international network specifically addressing vision impairments does not yet exist.

To advance these structural changes in a sustainable and equitable way, close collaborations between researchers, policymakers, practicing clinicians and persons with disabilities are essential (Hardt, Graser, et al. 2025b). However, such progress must not be confined to national initiatives alone. Rather, it requires coordinated international efforts and shared standards, aligned with the CRPD (UN General Assembly 2007), which explicitly calls for inclusive training of healthcare professionals and the removal of systemic barriers. Cross‐sectoral and cross‐national alliances are thus critical to strengthening educational frameworks, fostering research‐informed guidelines and ensuring that high‐quality mental healthcare is accessible to persons with disabilities across Europe and beyond.

## 5 Conclusion

Overall, our study contributes to the growing body of evidence highlighting the relevance of disability‐related competencies in outpatient psychotherapy. It yields five key implications: (1) Outpatient psychotherapy for persons with disabilities remains significantly underdeveloped, despite high prevalence rates and generally favourable practitioner attitudes; (2) although psychotherapists express openness, they frequently lack adequate structural and curricular support; (3) there is an urgent need for comprehensive academic instruction, targeted continuing education and sustained professional resources in this field—particularly programmes tailored to specific types of impairments; (4) many of the barriers identified are systemic, yet the findings point to a strong professional readiness to address them; and (5) advancing inclusive psychotherapy requires shared responsibility among political, institutional and academic stakeholders. We hope that our findings will inform ongoing discussions and reform initiatives in research, education and health policy and contribute to a more evidence‐based and equitable provision of mental health services for persons with disabilities.

## Ethics Statement

The current study got approval from the Ethics Committee of Witten/Herdecke University (Ethics Application No. S‐231/2021) and was conducted in accordance with national law and the tenets of the Declaration of Helsinki of 1975 (as revised). Informed consent was obtained from all participants.

## Consent

The authors have nothing to report.

## Conflicts of Interest

The authors declare no conflicts of interest.

## Supporting information


**Supporting Information S1:** This document provides additional tables and figures referenced in the article.

**Supporting Information S2:** Supplemental Material_2 questionnaires.pdf.

## Data Availability

The datasets generated and analysed during the current study are not publicly available due to restrictions imposed by the data provider, the Association of Statutory Health Insurance Physicians North Rhine (Kassenärztliche Vereinigung Nordrhein, KVNO). Data are available from the first author upon reasonable request and with permission of the KVNO. The self‐developed questionnaire and additional tables and figures are provided as Supporting Information.

## References

[cpp70159-bib-0001] Alnahdi, G. H. , S. Woodcock , and W. Agbeke . 2020. “The Impact of Contact on Attitudes Toward Including Students With Disabilities in Saudi Arabian Schools.” International Journal of Disability, Development and Education 67, no. 3: 304–318. 10.1080/1034912X.2019.1626005.

[cpp70159-bib-0002] Besika, J. , E. Kourkoutas , and P. Kendeou . 2018. “Psychologists' and Special Educators' Attitudes Toward Individuals With Intellectual Disabilities: The Role of Training and Professional Experience.” International Journal of Developmental Disabilities 64, no. 3: 140–152. 10.1080/20473869.2017.1332018.

[cpp70159-bib-0003] Bron, J. 2015. Psychotherapy and Mental Health for People With Intellectual Disabilities: A Handbook of Theory, Research and Practice. Routledge.

[cpp70159-bib-0004] Brunes, A. , M. B. Hansen , and T. Heir . 2019. “Post‐Traumatic Stress Reactions Among Individuals With Visual Impairments: A Systematic Review.” Disability and Rehabilitation 41, no. 18: 2111–2118. 10.1080/09638288.2018.145988. 29644887

[cpp70159-bib-0005] Buckles, J. , R. Luckasson , and E. Keefe . 2013. “A Systematic Review of the Prevalence of Psychiatric Disorders in Adults With Intellectual Disability, 2003–2010.” Journal of Mental Health Research in Intellectual Disabilities 6, no. 3: 181–207. 10.1080/19315864.2011.651682.

[cpp70159-bib-0006] Chacón, F. , A. Barrón , A. Aluja , and J. Fernández‐Castro . 2013. “Attitudes Toward People With Disabilities: A Study of Psychotherapists.” Journal of Intellectual & Developmental Disability 38, no. 3: 245–254. 10.3109/13668250.2013.804884.

[cpp70159-bib-0007] Council of the European Union . 2023. The Situation of Persons With Disabilities in the European Union: Progress Report 2023. Council of the European Union. https://www.consilium.europa.eu/.

[cpp70159-bib-0008] Critchfield, A. B. 2002. “Addressing the Needs of Deaf and Deafblind Clients in Psychotherapy.” American Annals of the Deaf 147, no. 3: 19–29. 10.1037/pro0000373.

[cpp70159-bib-0009] Critchfield, A. B. , and K. Ehlers . 2018. “Best Practices in Psychotherapy With Clients Who Are Deaf or Hard of Hearing.” Professional Psychology: Research and Practice 49, no. 4: 271–278. 10.1037/pro0000373.

[cpp70159-bib-0010] Deutscher Bundestag . 2019. “Gesetz über den Beruf des Psychotherapeuten (Psychotherapeutengesetz ‐ PsychThG).” Bundesgesetzblatt, Teil I (33), 1354–1368. Retrieved from https://www.bgbl.de.

[cpp70159-bib-0011] Dovidio, J. F. , L. Pagotto , and M. R. Hebl . 2011. “Implicit Attitudes and Biases in Psychotherapy.” Journal of Social Issues 67, no. 3: 436–449. 10.1111/j.1540-4560.2011.01701.x.

[cpp70159-bib-0012] Erceg‐Hurn, D. M. , and V. M. Mirosevich . 2008. “Modern Robust Statistical Methods: An Easy Way to Maximize the Accuracy and Power of Your Research.” American Psychologist 63, no. 7: 591–601. 10.1037/0003-066X.63.7.591. 18855490

[cpp70159-bib-0013] European Disability Forum . 2022. Breaking Barriers: Accessibility and Inclusion in Mental Health Services. European Disability Forum Retrieved from https://www.edf‐feph.org.

[cpp70159-bib-0015] European Society for Mental Health and Deafness n.d. Our vision https://esmhd.org/our‐vision.

[cpp70159-bib-0017] Glickman, N. 2008. Cognitive‐Behavioral Therapy for Deaf and Hearing Persons With Language and Learning Challenges. Taylor & Francis.

[cpp70159-bib-0018] Graser, J. , J. Göken , N. Lyons , T. Ostermann , and J. Michalak . 2022. “Cognitive‐Behavioral Therapy for Adults With Intellectual Disabilities: A Meta‐Analysis.” Clinical Psychology: Science and Practice 29, no. 3: 227–242. 10.1037/cps0000077.

[cpp70159-bib-0019] Hardt, B. , T. Heidenreich , C. Hunger‐Schoppe , et al. 2025a. “Barriers and Accessibility‐Improving Strategies in Mental Health Services for Persons With Hearing or Vision Impairments: Perspectives From Professionals and Clients—A Qualitative Interview Study.” Psychology and Psychotherapy: Theory, Research and Practice Advance online publication. 10.1111/papt.70006. PMC12905524 40801416

[cpp70159-bib-0020] Hardt, B. , J. Graser , T. Heidenreich , and J. Michalak . 2025b. “A Systematic Review and Meta‐Analysis of Psychological Interventions for Persons With Hearing or Vision Impairment: Research Gaps and Call to Action.” Clinical Psychology: Science and Practice 32, no. 2: 135–154. 10.1037/cps0000229.

[cpp70159-bib-0021] Hatton, C. , J. Rose , and E. Emerson . 2021. “Therapeutic Challenges When Working With People With Intellectual Disabilities: Communication, Emotional Regulation, and Relational Difficulties.” Journal of Applied Research in Intellectual Disabilities 34, no. 5: 1060–1072. 10.1111/jar.12879.

[cpp70159-bib-0022] Inclusion Europe . 2021. Universal Design and Mental Health Services: A Report on Accessibility for People With Intellectual Disabilities. Inclusion Europe Retrieved from https://www.inclusion‐europe.eu.

[cpp70159-bib-0023] International Association for the Scientific Study of Intellectual and Developmental Disabilities (IASSIDD) . n.d. “Home.” https://www.iassidd.org/.

[cpp70159-bib-0024] Jiang, F. , C. Kubwimana , J. Eaton , H. Kuper , and T. Bright . 2020. “The Relationship Between Mental Health Conditions and Hearing Loss in Low‐ and Middle‐Income Countries.” Tropical Medicine & International Health 25, no. 6: 646–659. 10.1111/tmi.13393. 32219942

[cpp70159-bib-0025] Kassenärztliche Bundesvereinigung [National Association of Statutory Health Insurance Physicians] 2024 Participants in Outpatient Care: 2023 Year‐End Statistics https://gesundheitsdaten.kbv.de/cms/html/16393.php.

[cpp70159-bib-0026] Kassenärztlichevereinigung Nordrhein, KVNO [Association of Statutory Health Insurance Physicians and Psychotherapists of Northrhein] . n.d. Bedarfsplanung. KV Nordrhein. https://www.kvno.de/praxis/niederlassung‐kooperation/bedarfsplanung.

[cpp70159-bib-0027] Mazza, M. G. , A. Rossetti , G. Crespi , and M. Clerici . 2020. “Prevalence of Co‐Occurring Psychiatric Disorders in Adults and Adolescents With Intellectual Disability: A Systematic Review and Meta‐Analysis.” Journal of Applied Research in Intellectual Disabilities 33, no. 2: 126–138. 10.1111/jar.12654. 31430018

[cpp70159-bib-0028] Mental Health Europe . 2020. Guide for Mental Health Professionals: Ensuring Accessibility and Inclusion for Persons With Disabilities. MHE Retrieved from https://www.mhe‐sme.org.

[cpp70159-bib-0029] OECD . 2022. Health at a Glance: Europe 2022—State of Health in the EU Cycle. Organisation for Economic Co‐operation and Development. 10.1787/4dd50c09-en.

[cpp70159-bib-0030] Olkin, R. 2017. Disability‐Affirmative Therapy: A Case Formulation Template for Clients With Disabilities. Oxford University Press.

[cpp70159-bib-0031] Olkin, R. , H. S. Hayward , and C. Abbott . 1996. “Psychologists' Experiences Working With Clients With Disabilities.” Professional Psychology: Research and Practice 27, no. 5: 429–437. 10.1037/0735-7028.27.5.429.

[cpp70159-bib-0032] Page, A. S. , and M. R. Islam . 2015. “The Role of Contact, Culture, and Social Norms in Shaping Attitudes Toward People With Disabilities: Evidence From a Cross‐National Study.” Journal of Social Issues 71, no. 3: 488–507. 10.1111/josi.12124.

[cpp70159-bib-0033] Salvador‐Carulla, L. , R. Martínez‐Leal , C. Heyler , M. R. Gutiérrez‐Colosía , C. Giné , and R. Nuño . 2013. “Training on Intellectual Disability in Health Sciences: The European Perspective.” International Journal of Developmental Disabilities. 10.1179/2047387713Y.0000000027. PMC4334381 25705375

[cpp70159-bib-0044] Schröter, A. , S. Schulze , K. Krause , and J. Kuhl . 2019. “Entwicklung und Validierung des EXPE‐B: Ein Fragebogen zur Messung der expliziten Einstellungen zu Behinderung.” Vierteljahresschrift für Heilpädagogik und ihre Nachbargebiete 88, no. 4: 304–319. 10.2378/vhn2019.art43d.

[cpp70159-bib-0042] Shoham, N. , G. Lewis , G. Favarato , and C. Cooper . 2019. “Prevalence of Anxiety Disorders and Symptoms in People With Hearing Impairment: A Systematic Review.” Social Psychiatry and Psychiatric Epidemiology 54: 649–660. 10.1007/s00127-018-1638-3. 30547211

[cpp70159-bib-0034] Shields, N. , and N. F. Taylor . 2014. “Contact With Young Adults With Disability Led to a Positive Change in Attitudes Toward Disability Among Physiotherapy Students.” Physiotherapy Canada 66, no. 3: 298–305. 10.3138/ptc.2013-56. 25125784 PMC4130409

[cpp70159-bib-0035] Statistisches Bundesamt [Federal Statistical Office of Germany] . 2024. 7,9 Millionen Schwerbehinderte Menschen Leben in Deutschland. https://www.destatis.de/DE/Presse/Pressemitteilungen/2024/07/PD24_281_227.html.

[cpp70159-bib-0036] Steinlechner, J. , N. Rüsch , and P. W. Corrigan . 2017. “Accessibility of Psychotherapy Practices for People With Disabilities in Germany.” Psychotherapy Research 27, no. 4: 457–465. 10.1080/10503307.2015.1114687.

[cpp70159-bib-0037] Taherdoost, H. , and M. Madanchian . 2024. “The Impact of Survey Response Rates on Research Validity and Reliability.” In Design and Validation of Research Tools and Methodologies, 183–212. 10.4018/979-8-3693-1135-6.ch009.

[cpp70159-bib-0038] UN General Assembly . 2007. “Convention on the Rights of Persons With Disabilities: Resolution / Adopted by the General Assembly, 24 January 2007, A/RES/61/106.”

[cpp70159-bib-0039] Wilcox, R. R. 2017. Introduction to Robust Estimation and Hypothesis Testing. 4th ed. Academic Press.

[cpp70159-bib-0040] World Health Organization . 2001. International Classification of Functioning, Disability and Health (ICF). World Health Organization.

[cpp70159-bib-0041] World Health Organization . n.d. Disability. Retrieved January 7, 2025, from https://www.who.int/health‐topics/disability.

[cpp70159-bib-0043] Zheng, Y. , X. Wu , X. Lin , and H. Lin . 2017. “The Prevalence of Depression and Depressive Symptoms Among Eye Disease Patients: A Systematic Review and Meta‐Analysis.” Scientific Reports 7, no. 46453. 10.1038/srep46453. PMC5388862 28401923

