# Supplementary material for: Psychotherapists' Perspectives and Support Needs in Treating Patients With Disabilities: Results From an Online Survey
Source: Clin Psychol Psychother. 2025 Sep 30;32(5):e70159. doi: 10.1002/cpp.70159 (PMC12484398; doi:10.1002/cpp.70159)
Supplement: Supplementary file 1 — Supporting Information S1: This document provides additional tables and figures referenced in the article. [file CPP-32-e70159-s002.pdf]

Supplemental Material 1

This document provides additional tables and figures referenced in the article.

Supplementary Table 1

Sample characteristics including demographic data and reported therapy approaches

	**female** *n* **= 286 (77.1%)**	**male** *n* **= 83 (22.4%)**	**diverse** *n* **= 2 (0.5%)**	**Total** *N* **= 371 (100%)**
Mean age (*SD*)	51.34 (10.44)	57.87 (9.8)	47 (12.73)	52.78 (10.64)
Range	30-81	34-78	38-56	30-81
Therapists background:				
Psychologists	217 (75.9%)	62 (74.7%)	1 (50%)	280 (75.5%)
Physicians	69 (24.1%)	22 (26.5%)	1 (50%)	92 (24.8%)
Personal disability	18 (6.3%)	5 (6%)	NM	23 (6.3%)
Contact to persons with disabilities in personal life	95 (33.2%)	28 (33.7%)	NM	123 (33.2%)
Therapy approach:				
Cognitive behavioral therapy	178 (62.2%)	49 (59%)	1 (50%)	228 (61.5%)
Psychodynamic Psychotherapy	118 (41.3%)	35 (42.2%)	1 (50%)	154 (41.5%)
Psychoanalysis	27 (9.4%)	8 (9.6%)	NM	35 (9.4%)
Systemic Psychotherapy	13 (4.5%)	12 (14.5%)	NM	25 (6.7%)
Additional approaches	34 (11.9%)	14 (16.9%)	NM	48 (12.9%)

Note: NM = Not Mentioned.

Supplementary Table 2

Treatment frequency of patients *with and without disabilities*

	**Patients with disabilities (total career)**	**Patients with disabilities (per year)**	**Patients without Disabilities (per year)**	**% of patients with disabilities (per year)**	**Total number of patients (per year)**
All data	n = 338	*n* = 339	*n* = 338	*n* = 338	*n* = 338
M (*SD*)	82.77 (337.48)	10.90 (30.16)	120.14 (243.61)	7.92 (12.09)	130.48 (260.60)
Range	1 – 5,000	0 – 300	.5 – 4,000	0 – 99.36	1 – 4,250
Mdn	12	3	70	3.85	80
≤ 95th Pctl.	*n* = 322	*n* = 322	*n* = 322	*n* = 322	*n* = 322
M (*SD*)	31.29 (55)	6.32 (9.38)	88.05 (61.80)	5.7 (5.8)	96.02 (67.89)
Mdn	10	2	70	3.73	77
Range	1 – 400	0 – 50	.5 – 300	0 – 30.77	1 – 350

Note: ≤ 95th Pctl. = represents data restricted to the 95th percentile.

Supplementary Table 3

Information needs regarding further training varied in types of impairment, including mean differences in the EXPE-B attitude score (independent-sample t-test)

	**Need for further training (*n*; %)**	**EXPE-B attitude score *M* (*SD*)**	***t*(*df*)**	***p***	***d***
Physical impairment	Yes (*n* = 120; 32.3%)	70.81 (7.45)	-1.73 (369)	.084	-.19
	No (*n* = 251; 67.7%)	69.38 (7.41)			
Intellectual impairment	Yes (*n* = 221; 59.6%)	70.87 (7.12)	-3.26 (369)	.001	-.35
	No (*n* = 150; 40.4%)	69.33 (7.67)			
Hearing impairment	Yes (*n* = 212; 57.1%)	70.93 (7.09)	-3.30 (369)	.001	-.35
	No (*n* = 159; 42.9%)	68.39 (7.68)			
Vision impairment	Yes (*n* = 156; 42.0%)	70.56 (7.29)	-1.59 (369)	.112	-.17
	No (*n* = 215; 58.0%)	69.32 (7.53)			
No need for further training	Yes (*n* = 75; 20.2%)	67.21 (8.17)	3.48 (369)	< .001	.45
	No (*n* = 296; 79.8%)	70.51 (7.11)			

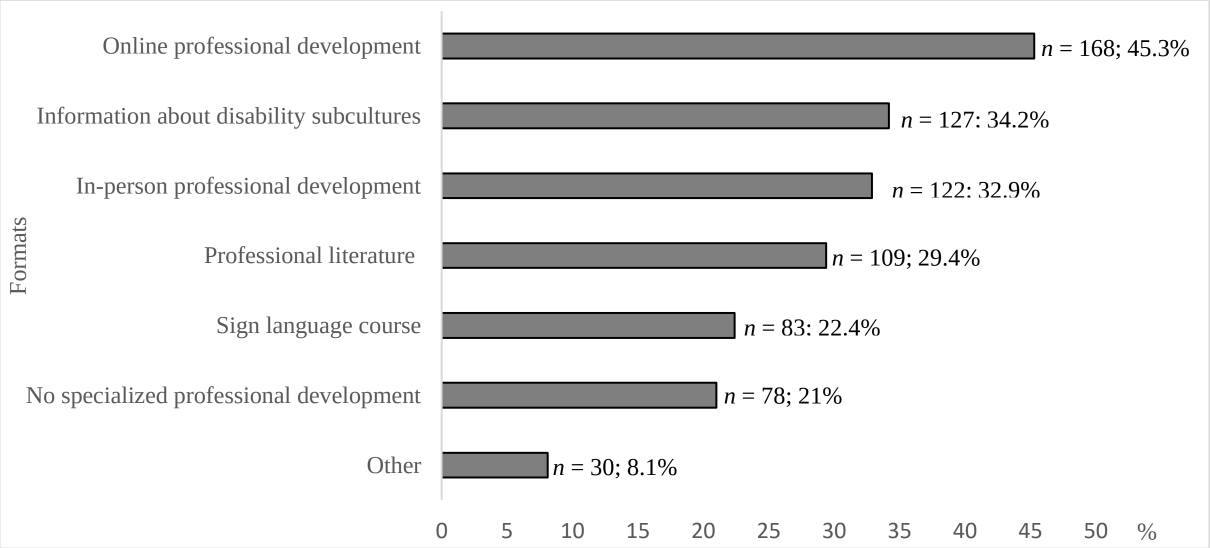

Supplementary Figure 1

Number and percentage of reported formats for professional development
