# Supplementary material for: Psychotherapists' Perspectives and Support Needs in Treating Patients With Disabilities: Results From an Online Survey
Source: Clin Psychol Psychother. 2025 Sep 30;32(5):e70159. doi: 10.1002/cpp.70159 (PMC12484398; doi:10.1002/cpp.70159)
Supplement: Supplementary file 2 — Supporting Information S2: Supplemental Material_2 questionnaires.pdf. [file CPP-32-e70159-s001.pdf]

Supplemental Material 2

All instruments were originally administered in German. Items were translated into English by the research team for the purpose of this supplemental file. The translation aimed to preserve semantic equivalence but has not undergone formal validation. Where relevant, back-translation or expert consultation was used to ensure clarity.

Section

Self-developed questionnaire (German) 2
Self-developed questionnaire (English) 8
EXPE-B, Schröter et al. (2019) (German) 14
EXPE-B, Schröter et al. (2019) (English) 16
Letter to members of the KVNO (German) 18
Letter to members of the KVNO (English) 19
Participant Information and Declaration of Consent (German) 20
Participant Information and Declaration of Consent (English) 24

Self-developed questionnaire (German)

Supplementary Table 1

Self-developed questionnaire in German (original version)

Nr.	Item	Scale
1	Wie alt sind Sie?	Angabe Alter in Jahren
2	Welchem Geschlecht fühlen Sie sich zugehörig?	• Männlich<br>• Weiblich<br>• Divers<br>• Andere (freie Eingabe)
3	Wie lautet Ihre aktuelle Berufsbezeichnung?	• Psychologische(r) Psychotherapeut*in<br>• Ärztliche (r) Psychotherapeut*in
4	In welchen psychotherapeutischen Therapieformen sind Sie ausgebildet?	• Verhaltenstherapie<br>• Systemische Therapie<br>• Tiefenpsychologisch fundierte Psychotherapie<br>• Psychoanalytische fundierte Psychotherapie<br>• Zusätzlich: (freie Eingabe)
5	Haben Sie einen eingetragenen Grad der Behinderung?	• Ja<br>• Nein

6	Leben Menschen mit einer Behinderung in Ihrem unmittelbaren persönlichen Umfeld?	• Ja<br>• Nein
7	Haben Sie in Ihrem beruflichen Kontext als Psychotherapeut*in bereits Erfahrungen mit Patient*innen mit körperlichen, intellektuellen oder sinnesbedingten Beeinträchtigungen gemacht	• Ja<br>• Nein
8	Wenn ja: Welche Beeinträchtigungen haben Sie bisher als Psychotherapeut*in kennengelernt?	• Körperliche Beeinträchtigungen<br>• Intellektuelle Beeinträchtigungen<br>• Hörbeeinträchtigungen<br>• Sehbeeinträchtigungen
9	Wie würden Sie Ihre generellen Erfahrungen mit Menschen mit Behinderung im Kontext der Psychotherapie beschreiben?	• Sehr positive<br>• eher positive<br>• gemischt<br>• eher negative<br>• durchweg negativ
10	Wie beschreiben Sie Ihre bisherigen Erfahrungen mit Menschen mit Behinderung in der Psychotherapie bezogen auf die folgenden Beeinträchtigungen:<br>• Körperliche Beeinträchtigungen<br>• Intellektuelle Beeinträchtigungen<br>• Hörbeeinträchtigungen<br>• Sehbeeinträchtigungen	• Sehr positive<br>• eher positive<br>• gemischt<br>• eher negative<br>• durchweg negativ<br>• bisher keine Erfahrung

11	Wie viele Menschen mit Behinderung haben Sie in Ihrer gesamten Laufbahn als Psychotherapeut*in etwa behandelt?	Angabe in Zahlen
12	Wie viele Patient*innen mit Beeinträchtigungen haben Sie schätzungsweise in einem Jahr?	Gesamtzahl Patient*innen ohne Beeinträchtigung und Anzahl Patient*innen mit Beeinträchtigung (Angabe in Zahlen)
13	Würden Sie sich fachliche Unterstützung im beruflichen Umgang mit Menschen mit Behinderung wünschen?	• Ich wünsche mir auf jeden Fall Unterstützung<br>• Ich wünsche mir eher Unterstützung<br>• teils, teils<br>• Ich wünsche mir eher keine Unterstützung<br>• Ich wünsche mir gar keine Unterstützung
14	Wie barrierefrei würden Sie Ihr eigenes Arbeitsumfeld als Psychotherapeut*in prinzipiell einschätzen?	• Voll und ganz barrierefrei<br>• eher barrierefrei<br>• teils, teils<br>• eher nicht barrierefrei<br>• überhaupt nicht barrierefrei
15	Wie barrierefrei würden Sie Ihr eigenes Arbeitsumfeld als Psychotherapeut*in mit Bezug auf die folgenden Beeinträchtigungsarten einschätzen? Bitte wählen Sie die zutreffende Antwort für jeden Punkt aus:<br>• Körperliche Beeinträchtigungen<br>• Intellektuelle Beeinträchtigungen<br>• Hörbeeinträchtigungen<br>• Sehbeeinträchtigungen	• Voll und ganz barrierefrei<br>• eher barrierefrei<br>• teils, teils<br>• eher nicht barrierefrei<br>• überhaupt nicht barrierefrei

16	Wo sehen Sie Barrieren für Menschen mit Behinderung in Ihrem persönlichen Arbeitsumfeld als Psychotherapeut*in? Mehrfaches Antworten ist möglich.	• Barrieren bei der Kontaktaufnahme (Zum Beispiel: Erreichbarkeit per Telefon, SMS, FAX, EMail, etc.)<br>• Barrieren in der non verbalen und/oder verbalen Kommunikation (Zum Beispiel: das Fehlen einer induktiven Höranlage)<br>• Barrieren in Ihrem Gebäude (Zum Beispiel: Barrieren durch Treppen oder zu enge Gänge oder Türen, Toilettenräume, Akustik im Gebäude, etc.)<br>• Barrieren auf dem Weg zur Therapie (Zum Beispiel: keine ÖPNV-Anbindung, Parkmöglichkeiten, Behindertenparkplätze, etc.)<br>• Barrieren innerhalb der Therapiesitzung (Zum Beispiel: Verständnisschwierigkeiten in Einzel oder Gruppenangeboten, Durchführung von Diagnostik, Bearbeitung von Therapie Material, Aufgaben oder Übungen in der Therapie, etc.)<br>• Ich sehe keine Barrieren in meinem persönlichen Arbeitsumfeld als Psychotherapeut*in<br>• Sonstiges: freie Eingabe
17	Wie barrierefrei würden Sie die ambulante psychotherapeutische Versorgung für Menschen mit Behinderung in Deutschland beurteilen?	• Voll und ganz barrierefrei<br>• eher barrierefrei<br>• teils, teils<br>• eher nicht barrierefrei<br>• überhaupt nicht barrierefrei
18	Würden Sie den Behandlungsaufwand von Menschen mit Behinderung höher einschätzen als bei Menschen ohne Behinderung?	• Ich schätze den Behandlungsaufwand deutlich höher ein<br>• Ich schätze den Behandlungsaufwand eher höher ein<br>• teils, teils<br>• Ich schätze den Behandlungsaufwand eher nicht höher ein

		• Ich schätze den Behandlungsaufwand gar nicht höher ein
19	Wie beurteilen Sie die vorherige Frage des Behandlungsaufwands auf die bereits erwähnten vier Arten von Beeinträchtigungen? Bitte wählen Sie die zutreffende Antwort für jeden Punkt aus:<br>• Körperliche Beeinträchtigungen<br>• Intellektuelle Beeinträchtigungen<br>• Hörbeeinträchtigungen<br>• Sehbeeinträchtigungen	• Ich schätze den Behandlungsaufwand deutlich höher ein<br>• Ich schätze den Behandlungsaufwand eher höher ein<br>• teils, teils<br>• Ich schätze den Behandlungsaufwand eher nicht höher ein<br>• Ich schätze den Behandlungsaufwand gar nicht höher ein.
20	Wie umfangreich würden Sie Ihr Wissen über Menschen mit Behinderung einschätzen. Bitte ordnen Sie sich einer der folgenden Gruppen zu. „Gruppe 1“entspricht dabei der Gruppe mit dem größten Wissen, „Gruppe 2“ mit dem zweitgrößten und „Gruppe 3“ mit dem drittgrößten Wissensstand	• Gruppe 1 - Expertenwissen: Ich habe mich als Psychotherapeut*in mit verschiedenen Arten von Beeinträchtigungen fachlich auseinandergesetzt und habe mir im Kontakt ein Bild über die Lebensumstände der Betroffenen machen können<br>• Gruppe 2 - Fachwissen: Ich greife nur auf Kenntnisse zurück, die mir aus meiner Ausbildung, Studium, Schulzeit und Allgemeinwissen zur Verfügung stehen<br>• Gruppe 3 - Grundwissen: Ich habe mich mit der Thematik kaum bis gar nicht auseinandergesetzt und kann mich nicht daran erinnern, in meiner Ausbildung, Studium, Schulzeit etwas darüber gelernt zu haben. So beziehe ich mich größtenteils nur auf mein Allgemeinwissen
21	Wurde das Thema Menschen mit Behinderung in Ihrer Ausbildung zum/zur Psychotherapeut*in aufgegriffen?	• Ja, es wurde ausführlich besprochen<br>• Ja, es wurde kurz erwähnt<br>• Nein, es wurde nicht erwähnt<br>• Ich weiß es nicht mehr

22	Wurde das Thema Menschen mit Behinderung in Ihrem Studium aufgegriffen?	• Ja, es wurde ausführlich besprochen<br>• Ja, es wurde kurz erwähnt<br>• Nein, es wurde nicht erwähnt<br>• Ich weiß es nicht mehr
23	Zu welchen Arten an Beeinträchtigungen würden Sie sich mehr Aufklärung bzw. Weiterbildung wünschen? Mehrfaches Antworten ist möglich.	• zu körperlichen Beeinträchtigungen<br>• zu intellektuellen Beeinträchtigungen<br>• zu Hörbeeinträchtigungen<br>• zu Sehbeeinträchtigungen<br>• Ich wünsche mir keine weitere Aufklärung bzw. Weiterbildung
24	Welche Art von Unterstützung würden Sie sich bezüglich des Themas Barrierefreiheit in der Psychotherapie wünschen? Mehrfaches Antworten ist möglich.	• Online-Weiterbildungen, um mehr Informationen über Menschen mit Behinderung in der Psychotherapie und Barrierefreiheit in der Psychotherapie zu erhalten<br>• Weiterbildungen in Präsenz (z.B. Informationstage), um mehr Informationen über Menschen mit Behinderung in der Psychotherapie und Barrierefreiheit in der Psychotherapie zu erhalten<br>• Literatur zum Thema Menschen mit Behinderung als Patient*innen in der Psychotherapie<br>• Zugang zu einem Kurs in Gebärdensprache/Informationen zu Subkulturen von Menschen mit Behinderung, wie z.B. der Gehörlosenkultur<br>• Ich wünsche mir keine Art von Unterstützung<br>• Sonstiges: freie Eingabe

Self-developed questionnaire (English)

Supplementary Table 2

Self-developed questionnaire in English (translated version)

Nr.	Item	Scale
1	How old are you?	*Please indicate your age in years.*
2	Which gender do you identify with?	• Male<br>• Female<br>• Diverse<br>• Other: (free text)
3	What is your current professional title?	• Psychotherapist (psychological)<br>• Psychotherapist (medical)
4	Which psychotherapeutic approaches are you trained in?	• Cognitive behavioral therapy (CBT)<br>• Systemic therapy<br>• Psychodynamic therapy<br>• Psychoanalytic therapy<br>• Additional: (free text)
5	Do you have a registered degree of disability?	• Yes<br>• No

6	Do persons with disabilities live in your immediate personal environment?	• Yes<br>• No
7	Have you had professional experience as a psychotherapist with patients who have physical, intellectual, or sensory impairments?	• Yes<br>• No
8	If yes: What types of impairments have you encountered in your work as a psychotherapist?	• Physical impairments<br>• Intellectual impairments<br>• Hearing impairments<br>• Visual impairments
9	How would you describe your general experience with persons with disabilities in the context of psychotherapy?	• Very positive<br>• Rather positive<br>• Mixed<br>• Rather negative<br>• Very negative
10	How would you describe your previous experience with persons with disabilities in psychotherapy with regard to the following types of impairments:<br>• Physical impairments<br>• Intellectual impairments<br>• Hearing impairments<br>• Visual impairments	• Very positive<br>• Rather positive<br>• Mixed<br>• Rather negative<br>• Very negative<br>• No experience so far
11	Approximately how many persons with disabilities have you treated over the course of your psychotherapeutic career?	Please enter a number.

12	Approximately how many patients with impairments do you treat in a typical year?	Please enter the total number of patients without impairments and the number of patients with impairments.
13	Would you wish for professional support in your work with persons with disabilities?	• I definitely wish for support<br>• I would rather wish for support<br>• Partly yes, partly no<br>• I would rather not wish for support<br>• I definitely do not wish for support
14	How would you generally assess the accessibility of your own work environment as a psychotherapist?	• Fully accessible<br>• Rather accessible<br>• Partly accessible / partly inaccessible<br>• Rather not accessible<br>• Not accessible at all
15	How would you assess the accessibility of your own work environment as a psychotherapist with regard to the following types of impairments? Please select the appropriate response for each:<br>• Physical impairments<br>• Intellectual impairments<br>• Hearing impairments<br>• Visual impairments	• Fully accessible<br>• Rather accessible<br>• Partly accessible / partly inaccessible<br>• Rather not accessible<br>• Not accessible at all
16	Where do you see barriers for persons with disabilities in your personal work environment as a psychotherapist? Multiple answers possible.	• Barriers in initial contact (e.g., not accessible via phone, SMS, fax, email, etc.)<br>• Barriers in non-verbal and/or verbal communication (e.g., lack of an induction hearing loop)

		• Barriers in your building (e.g., stairs, narrow hallways or doors, restroom facilities, acoustics, etc.)<br>• Barriers on the way to therapy (e.g., no access to public transport, parking availability, lack of accessible parking spaces, etc.)<br>• Barriers during the therapy session (e.g., difficulties in understanding in individual or group sessions, conducting diagnostics, working with therapy materials, tasks or exercises, etc.)<br>• I do not see any barriers in my personal work environment as a psychotherapist<br>• Other: (free text)
17	How would you assess the accessibility of outpatient psychotherapeutic care for persons with disabilities in Germany?	• Fully accessible<br>• Rather accessible<br>• Partly accessible / partly inaccessible<br>• Rather not accessible<br>• Not accessible at all
18	Would you consider the treatment effort for persons with disabilities to be higher than for persons without disabilities?	• I consider the treatment effort to be significantly higher<br>• I rather consider the treatment effort to be higher<br>• Partly yes, partly no<br>• I rather do not consider the treatment effort to be higher<br>• I do not consider the treatment effort to be higher at all

19	How would you assess the previous question regarding treatment effort with respect to the following four types of impairments? Please select the appropriate response for each:<br>• Physical impairments<br>• Intellectual impairments<br>• Hearing impairments<br>• Visual impairments	• I consider the treatment effort to be significantly higher<br>• I rather consider the treatment effort to be higher<br>• Partly yes, partly no<br>• I rather do not consider the treatment effort to be higher<br>• I do not consider the treatment effort to be higher at all
20	How would you rate your level of knowledge about persons with disabilities? Please assign yourself to one of the following groups. "Group 1" represents the highest level of knowledge, "Group 2" the second-highest, and "Group 3" the lowest level.	• Group 1 – Expert knowledge: As a psychotherapist, I have engaged professionally with various types of impairments and have gained insight into the living conditions of affected persons through direct contact.<br>• Group 2 – Professional knowledge: My knowledge is based solely on what I learned during my training, university studies, school education, and general knowledge.<br>• Group 3 – Basic knowledge: I have dealt with the topic little or not at all and do not recall learning about it during my training, studies, or school years. My understanding is therefore largely based on general knowledge.
21	Was the topic of persons with disabilities addressed during your training as a psychotherapist?	• Yes, it was discussed in detail<br>• Yes, it was briefly mentioned<br>• No, it was not mentioned<br>• I don’t remember
22	Was the topic of persons with disabilities addressed during your university studies?	• Yes, it was discussed in detail

		• Yes, it was briefly mentioned<br>• No, it was not mentioned<br>• I don't remember
23	For which types of impairments would you wish for more information or further training? Multiple answers possible.	• On physical impairments<br>• On intellectual impairments<br>• On hearing impairments<br>• On visual impairments<br>• I do not wish for any further information or training
24	What kind of support would you wish for regarding the topic of accessibility in psychotherapy? Multiple answers possible.	• Online training to gain more information about persons with disabilities in psychotherapy and about accessibility in psychotherapy<br>• In-person training (e.g., informational events) to gain more information about persons with disabilities in psychotherapy and about accessibility in psychotherapy<br>• Professional Literature on the topic of persons with disabilities as patients in psychotherapy<br>• Access to a sign language course<br>• Information about subcultures of persons with disabilities, such as Deaf culture<br>• I do not wish for any kind of support<br>• Other: (free text)

EXPE-B, Schröter et al. (2019) (German)

Supplementary Table 3

Original German Version oft he EXPE-B (Schröter et al., 2019)

Nr.	Item
1	Wir sollten Menschen mit Behinderung und Menschen ohne Behinderung in dieselbe Nachbarschaft integrieren.
2	Ich würde es gut finden, wenn mein Kind eine Einladung zu einer Geburtstagsparty annehmen würde, die für ein Kind mit einer Behinderung gegeben wird.
3	Ich würde mich freuen, wenn mein Kind Kinder mit Behinderung als enge Freund*innen hat.
4	Wenn ich mir als Vermieter*in meine Mieter*innen aussuchen könnte, würde ich nur an Menschen ohne Behinderung vermieten.
5	Menschen mit Behinderung können nicht dieselben Leistungen am Arbeitsplatz erbringen, wie Menschen ohne Behinderung.
6	Ich habe nichts dagegen, einen Film oder ein Theaterstück in Gesellschaft von Menschen mit Behinderung zu besuchen.
7	Gesetze, die von den Arbeitgeber*innen verlangen, Menschen mit Behinderungen nicht zu diskriminieren, verletzten die Rechte der/des Einzelnen, die/der mit Menschen mit Behinderung keinen Umgang haben will.

8	Wenn ein Kind mit Legasthenie (Lese-Rechtsschreib-Schwäche) eine längere Bearbeitungszeit bei Klassenarbeiten erhält, ist das ungerecht den anderen Kindern gegenüber.
9	Ich würde Menschen mit Behinderung lieber nicht zusammen mit meinen Freund*innen, die keine Behinderung haben, zum Abendessen einladen.
10	Die Integration von Menschen mit und ohne Behinderung am Arbeitsplatz brächte für beide Seiten einen Vorteil.
11	In Deutschland wird für Menschen mit Behinderung zu viel Aufwand betrieben.
12	Viele Forderungen von Menschen mit Behinderung sind überzogen.
13	Das Problem von Vorurteilen gegenüber Menschen mit Behinderung wird überbewertet.
14	Auch wenn Menschen mit Behinderung einen Grund haben, sich zu beklagen, würden Sie ihre Ziele eher erreichen, wenn sie geduldiger wären.
15	Ich wäre gern bereit, zu einem kompetenten Friseur zu gehen, der eine Behinderung hat.
16	Auch wenn die soziale Gleichstellung verwirklicht wäre, könnten Menschen mit Behinderung sich in sozialen Situationen dennoch nicht wie Menschen ohne Behinderung verhalten.

Note: Scale from 0 = “stimme vollkommen überein“ to 5 = “stimme gar nicht überein“.

EXPE-B, Schröter et al. (2019) (English)

Supplementary Table 4

English translation of the EXPE-B (Schröter et al., 2019)

Nr.	Item
1	We should integrate people with disabilities and people without disabilities into the same neighborhood.
2	I would be pleased if my child accepted an invitation to a birthday party hosted by a child with a disability.
3	I would be happy if my child had children with disabilities as close friends.
4	If I could choose my tenants as a landlord, I would only rent to people without disabilities.
5	People with disabilities cannot perform at the same level in the workplace as people without disabilities.
6	I have no objection to attending a movie or a theater performance in the company of people with disabilities.
7	Laws that require employers not to discriminate against people with disabilities violate the rights of individuals who do not want to interact with people with disabilities.
8	If a child with dyslexia (reading and writing difficulties) is given extended time on tests, it is unfair to the other children.
9	I would prefer not to invite people with disabilities to dinner together with my friends who do not have disabilities.

10.	The integration of people with and without disabilities in the workplace would be beneficial for both sides.
11	In Germany, too much effort is made for people with disabilities.
12	Many demands made by people with disabilities are exaggerated.
13	The issue of prejudice against people with disabilities is often exaggerated.
14	Even if people with disabilities have a reason to complain, they would be more likely to achieve their goals if they were more patient.
15	I would be glad to go to a skilled hairdresser with a disability.
16	Even if social equality were achieved, people with disabilities still wouldn't be able to behave like people without disabilities in social situations.

Note: Scale from 0 = "strongly agree" to 5 = "strongly disagree".

Letter to **members of the KVNO (German)**

Anschreiben an die KVNO-Mitglieder

Sehr geehrte psychologische und ärztliche Psychotherapeut*innen,
die Fakultät für Klinische Psychologie und Psychotherapie II an der Universität Witten/Herdecke führt zurzeit eine Onlinebefragung zum Thema „**Barrierefreiheit in der Psychotherapie für Menschen mit Behinderungen**" durch und lädt Sie herzlichst zu einer Teilnahme ein.
Es werden derzeit psychologische und ärztliche Psychotherapeut/Innen gesucht, die bereit sind eine Online-Befragung auszufüllen. Die Befragung dauert nicht länger als 20 Minuten. Mit einer Teilnahme an unserer Forschung würden Sie helfen, ein umfassendes Bild über den derzeitigen Stand der ambulanten Psychotherapie für Menschen mit Behinderung zu erhalten. Mögliche Erkenntnisse könnte die Forschung im Bereich barrierefreie Psychotherapie fördern bzw. voranbringen – auch innerhalb von Aus- und/oder Fortbildungsmaßnahmen der klinischen Psychologie.
Das Forschungsvorhaben wird gemeinsam mit Prof. Dr. Johannes Michalak und zwei Masterstudierenden der Universität Witten/Herdecke durchgeführt. Dabei werden wissenschaftliche Standards bzgl. der Anonymität der TeilnehmerInnen und der Schweigepflicht eingehalten.
Ihre Erkenntnisse und Eindrücke aus Ihrer psychotherapeutischen Arbeit sind sehr wertvoll und wir würden uns sehr freuen, wenn Sie sich die Zeit nehmen würden, den Online-Fragebogen auszufüllen. Wenn wir Ihr Interesse geweckt haben, folgen sie gerne diesem Link (___).
Wir bedanken uns im Voraus für Ihre Bemühungen

Mit freundlichen Grüßen,
Bastian Hardt, M.Sc. Psych.

Fakultät für Gesundheit (Department für Psychologie und Psychotherapie)
Lehrstuhl für Klinische Psychologie und Psychotherapie II
Universität Witten/Herdecke, Alfred-Herrhausen-Straße 44, 58455 Witten
E-Mail: bastian.hardt@uni-wh.de

Letter to members of the KVNO (English)

Dear Psychotherapists,

The Faculty of Clinical Psychology and Psychotherapy II at Witten/Herdecke University is currently conducting an online survey on the topic of *"Accessibility in Psychotherapy for Persons with Disabilities"*, and we warmly invite you to participate.

We are looking for psychological and medical psychotherapists who are willing to complete an online questionnaire. The survey will take no longer than 20 minutes. By taking part in our research, you would contribute to creating a more comprehensive understanding of the current state of outpatient psychotherapy for persons with disabilities. The findings may support the advancement of accessible psychotherapy and contribute to the development of training and continuing professional development in the field of clinical psychology.

This research project is being conducted in collaboration with Prof. Dr. Johannes Michalak and two Master's students from Witten/Herdecke University. All scientific standards concerning participant anonymity and confidentiality are strictly upheld.

Your insights and experiences from your psychotherapeutic practice are extremely valuable to us, and we would greatly appreciate it if you could take the time to complete the online questionnaire. If we have sparked your interest, please follow this link: (___).

We thank you in advance for your time and support.

Kind regards,

Bastian Hardt, M.Sc. Psych.

Faculty of Health (Department of Psychology and Psychotherapy)
Chair of Clinical Psychology and Psychotherapy II
Witten/Herdecke University
Alfred-Herrhausen-Straße 44
58455 Witten
Email: bastian.hardt@uni-wh.de

Participant Information and Declaration of Consent (German)

Probandenaufklärung und Einverständniserklärung

Sehr geehrte Teilnehmerinnen und Teilnehmer,

vielen herzlichen Dank für Ihr Interesse an dieser Online-Befragung!
Mit Ihrer Teilnahme helfen Sie uns, ein besseres Verständnis von barrierefreier Psychotherapie zu erlangen. Durch Ihre Angaben erhoffen wir uns zu ermitteln, was barrierefreie Psychotherapie zum jetzigen Zeitpunkt bedeutet und ob Angebote wie Weiterbildungen und Informationstage zum Thema barrierefreie Psychotherapie sowie Arbeit mit Menschen mit Behinderung von Ihrer Seite gewünscht sind. Unser primäres Ziel ist es die Barrierefreiheit innerhalb der psychotherapeutischen Versorgung zu fördern und zu verbessern. Um uns dabei zu unterstützen, ist es Ihrerseits nicht notwendig, dass Sie bisher mit Menschen mit Behinderung im psychotherapeutischen Kontext gearbeitet haben. Ihnen werden im Folgenden Fragen über Erfahrungen, Erwartungen und Kenntnisse über die Themen Menschen mit Behinderung sowie Barrierefreiheit gestellt. Zusätzlich dazu werden Ihnen auch Fragen über bekannte psychologische Konstrukte begegnen. Bei der Begrifflichkeit Menschen mit Behinderung stützen wir uns auf die Definition der UN-Behindertenrechtskonvention:

“Artikel 1: Zu den Menschen mit Behinderungen zählen Menschen, die langfristige körperliche, seelische, intellektuelle oder Sinnesbeeinträchtigungen haben, welche sie in Wechselwirkung mit verschiedenen Barrieren an der vollen, wirksamen und gleichberechtigten Teilhabe an der Gesellschaft hindern können.” (*UN-Behindertenrechtskonvention, UN-BRK 2008*)

Dabei wird sich diese Studie primär auf körperliche, sinnesbedingte und intellektuelle Beeinträchtigungen konzentrieren.

Unser Team setzt sich aus Wissenschaftler*innen und Master-Studierenden der Universität Witten/Herdecke (Lehrstuhl für klinische Psychologie und Psychotherapie II) zusammen. Leiter des Lehrstuhls und dieses Forschungsprojekts ist Prof. Dr. Johannes Michalak. Die

Projektorganisation und -koordination liegt bei Bastian Hardt, M.Sc. (Wissenschaftlicher Mitarbeiter und Doktorand).

Proband*innenaufklärung:

Die Teilnahme an dieser Studie ist **freiwillig.** Ihre Daten werden **anonym** gespeichert und vertraulich behandelt. Weiterhin müssen Sie an keiner Stelle Ihren Namen, Ihre Adresse oder Ihr Geburtsdatum angeben. Wenn Sie sich beim Ausfüllen der Fragen unwohl fühlen, können Sie jederzeit ohne Angaben von Gründen von der Studie zurücktreten (d. h. das Ausfüllen abbrechen). Beachten Sie, dass die bis dahin getätigten Eingaben gespeichert werden.

Die Dauer der Teilnahme an dieser Studie beträgt etwa **20-30 Minuten**. Eine Unterbrechung der Teilnahme ist leider nicht möglich.

Wer kann teilnehmen?

Sie können an dieser Studie teilnehmen (Einschlusskriterien), wenn Sie…

- mindestens 18 Jahre alt sind
- Den Titel bzw. Approbation psychologischer/e Psychotherapeut*in oder ärztlicher/e Psychotherapeut*in tragen
- Deutsch sprechen
- nach Approbation Erwachsene Menschen behandeln dürfen
- Sie derzeit im ambulanten Setting arbeiten oder als Psychotherapeut*in niedergelassen sind

Wir möchten Sie bitten, **von dieser Studie abzusehen (Ausschlusskriterien),** wenn Sie…

- Keine Ausbildung zum/zur psychologischen oder ärztlichen Psychotherapeut*in abgeschlossen haben
- Keine Approbation für die Behandlung von Erwachsenen
- Sie sich noch in der Ausbildung zum/zur psychologischen oder ärztlichen Psychotherapeut*in befinden

Vergütung

Es wird keine Vergütung an Teilnehmer*innen ausgezahlt. Sie haben am Ende der Studie die Möglichkeit, Fragen über das Forschungsprojekt zu stellen. Diese Möglichkeit schließt mit

ein, dass Sie über die Ergebnisse des Projekts informiert werden können, wenn Sie es wünschen.

Fragen, Kommentare, Beschwerden:

Wenn Sie Fragen, Kommentare, Anmerkungen oder Beschwerden bezüglich der Studie haben, wenden Sie sich bitte per E-Mail an uns (Bastian.Hardt@uni-wh.de).

Datenschutzinformation nach Art. 13 DSGVO

Diese Online-Umfrage der Universität Witten/Herdecke (UW/H) wird als Forschungsarbeit am Lehrstuhl für Klinische Psychologie und Psychotherapie II durchgeführt. Die inhaltliche Verantwortung obliegt Prof. Dr. Johannes Michalak. Konkreter Ansprechpartner ist Bastian Hardt, M. Sc (Wissenschaftlicher Mitarbeiter, Bastian.Hardt@uni-wh.de).

Die Verarbeitung der Umfragedaten dient ausschließlich dem Zweck der wissenschaftlichen Forschung. Es werden nur jene Angaben verarbeitet, die Sie uns über die Felder der Online-Studie direkt mitteilen. Verbindungsdaten, die im Hintergrund aus technischen Gründen zusätzlich von Ihrem Endgerät übermittelt werden, werden getrennt von den Forschungsdaten gespeichert. Diese werden nur für eine kurze Zeit verarbeitet (z. B. für Zwecke der Systempflege und Fehlerbehebung) und nur von den System-Administrator*innen eingesehen. Diese Daten sind grundsätzlich von der Verarbeitung zu Forschungszwecken ausgenommen. Die Umfragedaten werden nur so lange aufbewahrt, wie es zur Erfüllung des konkreten Forschungszwecks erforderlich ist. Dies dauert voraussichtlich 3 Jahre an. Nach Abschluss der Studie werden diese so anonymisierten Aufzeichnungen in den Datenbestand des Lehrstuhls für klinische Psychologie und Psychotherapie II der Universität Witten/Herdecke übernommen. Die Aufbewahrungsdauer der erhobenen Daten beträgt 10 Jahre. Eine darüberhinausgehende Verarbeitung personenbezogener Daten oder eine Übermittlung an Dritte außerhalb der UW/H ist nicht vorgesehen.

Rechtsgrundlage für die Verarbeitung der Forschungsdaten ist Ihre freiwillige Einwilligung nach Art. 6 (1) a DSGVO, die Sie durch Betätigen der Schaltfläche unten und dem Aufruf der ersten Seite der Online-Umfrage aktiv bekunden. Die zusätzlichen Hintergrunddaten verarbeitet die UW/H nach Art. 6 (1) f DSGVO im berechtigten Eigeninteresse, jedoch ausschließlich für Zwecke der Wartung ihrer Websysteme.

Sie können Ihre Einwilligung zur Verarbeitung Ihrer Umfragedaten ohne nachteilige Folgen für Sie jederzeit mit Wirkung für die Zukunft widerrufen. Wenden Sie sich dazu bitte an

Bastian Hardt (Bastian.Hardt@uni-wh.de). Bitte beachten Sie, dass ein Löschwunsch nur für solche Daten erfüllt werden kann, die Ihrer Person in eindeutiger Weise zugeordnet werden können. Weitere allgemeine Informationen zum UW/H-Datenschutz finden Sie unter https://www.uni-wh.de/datenschutz/. Dort finden Sie neben zusätzlichen Informationen zu den Betroffenenrechten auch detaillierte Angaben zu den Verbindungsdaten, die im Hintergrund gemeinsam mit den Umfragedaten an die UW/H übermittelt werden. Den Datenschutzbeauftragten der UW/H erreichen Sie unter datenschutz@uni-wh.de.

Einverständniserklärung

Bevor die eigentliche Umfrage beginnt, benötigen wir Ihre **Zustimmung** zu Folgendem:

- Ich weiß, dass meine Teilnahme an dieser Studie freiwillig ist und meine Daten zu wissenschaftlichen Zwecken gespeichert werden
- Ich habe den Text der Proband*innenaufklärung gelesen und verstanden
- Ich habe die Datenschutzinformation gelesen und verstanden
- Ich erfülle die oben genannten Einschlusskriterien

Ich willige in die Teilnahme ein und akzeptiere damit alle oben genannten Punkte (inkl. der Datenschutzerklärung). Mir ist bekannt, dass ich die Umfrage jederzeit und ohne Angabe von Gründen abbrechen kann.

✅ Ich stimme zu.

Participant Information and Declaration of Consent (English)

Dear Participants,

Thank you very much for your interest in this online survey!

By taking part, you help us gain a better understanding of accessible psychotherapy. With your responses, we hope to identify what accessible psychotherapy currently means and whether offerings such as training programs or informational events on accessible psychotherapy and working with persons with disabilities are of interest to professionals. Our primary goal is to support and improve accessibility within psychotherapeutic care.

You do not need prior experience working with persons with disabilities in a psychotherapeutic setting to participate. In the following, you will be asked about your experiences, expectations, and knowledge regarding persons with disabilities and accessibility. Additionally, the survey contains questions concerning established psychological constructs.

In using the term people with disabilities, we refer to the definition provided by the UN Convention on the Rights of Persons with Disabilities:

"Article 1: Persons with disabilities include those who have long-term physical, mental, intellectual or sensory impairments which in interaction with various barriers may hinder their full and effective participation in society on an equal basis with others."

(UN Convention on the Rights of Persons with Disabilities, CRPD, 2008)

This study primarily focuses on physical, sensory, and intellectual impairments.

The research team consists of researchers and Master's students from the Chair of Clinical Psychology and Psychotherapy II at Witten/Herdecke University. The project is led by Prof. Dr. Johannes Michalak. The project is organized and coordinated by Bastian Hardt, M.Sc. (Research Associate and PhD Student).

<u>Participant Information</u>

Participation in this study is voluntary. Your data will be stored anonymously and treated confidentially. You will not be asked to provide your name, address, or date of birth at any point. Should you feel uncomfortable while completing the questionnaire, you may withdraw

from the study at any time and without stating any reasons (i.e., by terminating the survey). Please note that data already entered up to that point will still be stored.

The duration of participation is approximately 20–30 minutes. Unfortunately, it is not possible to interrupt and resume the survey at a later time.

Who can participate?

You may participate in this study (inclusion criteria) if you:

- Are at least 18 years old
- Hold the license (Approbation) as a psychological or medical psychotherapist
- Speak German
- Are licensed to treat adult patients
- Are currently working in outpatient care or are in private practice

Please refrain from participating (exclusion criteria) if you:

- Have not completed training as a psychological or medical psychotherapist
- Do not hold a license to treat adult patients
- Are currently in training to become a psychological or medical psychotherapist

Compensation

There is no financial compensation for participation. At the end of the survey, you will have the opportunity to ask questions about the project. You may also request to receive information about the study's results.

Questions, Comments, Complaints

If you have any questions, comments, remarks, or complaints regarding the study, please feel free to contact us by email: Bastian.Hardt@uni-wh.de

Data Protection Information (pursuant to Art. 13 GDPR)

This online survey is conducted by Witten/Herdecke University (UW/H) as part of a research project at the Chair of Clinical Psychology and Psychotherapy II. The person responsible for the content is Prof. Dr. Johannes Michalak. The direct contact person is Bastian Hardt, M.Sc. (Research Associate, Bastian.Hardt@uni-wh.de). The data you provide will be processed exclusively for scientific research purposes. Only information that you explicitly submit via the questionnaire fields will be processed. Connection data transmitted automatically by your

device will be stored separately from the research data and processed only for a short period (e.g., for system maintenance or troubleshooting). These data are accessed only by system administrators and are not used for research purposes. Survey data will only be stored for as long as is necessary to achieve the stated research purpose, which is expected to be three years. After conclusion of the study, anonymized data will be transferred to the data archive of the Chair of Clinical Psychology and Psychotherapy II at UW/H and stored for a maximum of ten years. There is no plan to further process personal data or to share them with third parties outside UW/H.

The legal basis for data processing is your voluntary consent in accordance with Art. 6 (1) (a) GDPR, which you provide by clicking the button below and proceeding to the first page of the survey. The aforementioned background data are processed in accordance with Art. 6 (1) (f) GDPR based on UW/H's legitimate interest in maintaining its web systems.

You may withdraw your consent to the processing of your survey data at any time without negative consequences. To do so, please contact Bastian Hardt (Bastian.Hardt@uni-wh.de). Please note that data can only be deleted if they can be clearly linked to your person.

For more information on data protection at UW/H, please visit:
https://www.uni-wh.de/datenschutz/

There you will also find further details about your rights as a data subject and information on the connection data transmitted with the survey.

The university's data protection officer can be contacted at: datenschutz@uni-wh.de

Declaration of Consent

Before starting the survey, we ask for your confirmation of the following:

- I understand that my participation in this study is voluntary and that my data will be stored for scientific purposes.
- I have read and understood the participant information above.
- I have read and understood the data protection notice.
- I meet the inclusion criteria listed above.

✅ I consent to participate in this study and accept all of the above (including the data protection declaration). I understand that I may withdraw from the survey at any time without giving a reason.
